# Excessive Daytime Sleepiness among First to Fourth Year Undergraduate Students of a Medical College in Nepal: A Descriptive Cross-sectional Study

**DOI:** 10.31729/jnma.5297

**Published:** 2020-09-30

**Authors:** Kumar Roka, Sabina Khadka, Sanju Dahal, Meenakshi Yadav, Puja Thapa, Rubina K.C.

**Affiliations:** 1 Department of Internal Medicine, Shree Birendra Hospital, Chhauni, Kathmandu, Nepal; 2 Nepalese Army Institute of Health Sciences, Sanobharyang, Kathmandu, Nepal

**Keywords:** *Epworth Sleepiness Scale*, *excessive Daytime Sleepiness*, *medical students*, *Nepal*, *prevalence*

## Abstract

**Introduction:**

Excessive Daytime Sleepiness is a significant health problem among medical students worldwide which can impair their cognitive and academic performances. Our study aims to determine the prevalence of Excessive Daytime Sleepiness among the first to fourth year undergraduate students of the Nepalese Army Institute of Health Sciences-College of Medicine.

**Methods:**

Following the ethical approval from the Institutional Review Committee with registration no. 317, a descriptive cross-sectional study was conducted among the first to fourth year medical students of the Nepalese Army Institute of Health Sciences-College of Medicine from 4^th^ to 10^th^ August 2020. Two hundred and thirty-two students were selected for the study using the stratified random sampling technique. Epworth Sleepiness Scale was used to obtain data on daytime sleepiness among the study participants. The data were entered into Google spreadsheets and later analyzed. Point estimate at 95% Confidence Interval was calculated along with the frequency and proportion for binary data.

**Results:**

The prevalence of Excessive Daytime Sleepiness among the first to fourth year undergraduate students of the Nepalese Army Institute of Health Sciences-College of Medicine is found to be 67 (31.02%) at 95% Confidence Interval (24.85-37.19). It was found to be highly prevalent among the fourth year undergraduate medical students 20 (35.09%) and least prevalent among the first year students 13 (26.00%). Excessive Daytime Sleepiness was found to be slightly higher among females 23 (34.85%) than males 44 (29.33%).

**Conclusions:**

Excessive Daytime Sleepinessis highly prevalent among medical students in our study as suggested by various international studies.

## INTRODUCTION

International classification of sleep disorders defined Excessive Daytime Sleepiness (EDS) as difficulty in maintaining the alert awake state, usually accompanied by a rapid entrance into sleep when the person is sedentary.^1^ Heavy academic loads, excessive internet use at night, coffee intake especially at night, too much exposure to artificial lights, etc. increases the risk of EDS. Similarly, EDS might be associated with various sleep disorders such as sleep deprivation, obstructive sleep apnea, narcolepsy, idiopathic hypersomnia, insomnia, circadian rhythm disorders, etc.^1–4^

Even though EDS is a significant health problem, it hasn't been explored much among the medical university students especially in the Asian countries.^5–6^ To our knowledge, no studies on EDS has been conducted among the medical students in Nepal.

In this study, we aimed to determine the prevalence of EDS among the first to fourth year undergraduate students of the Nepalese Army Institute of Health Sciences-College of Medicine.

## METHODS

A descriptive cross-sectional study was conducted among the first to fourth year undergraduate medical students of the Nepalese Army Institute of Health Sciences-College of Medicine (NAIHS-COM) from 4^th^ to 10^th^ August 2020. The study was conducted after receiving ethical approval from the Institutional Review Committee (IRC), NAIHS on July 2020 with the Reg. No. 317. The sample size for the study was calculated using the following formula:

$$\begin{array}{ll} \text{n} & \text{= }{\text{Z}}^{\text{2}}\times \text{p}\times \text{q}/{\text{e}}^{\text{2}}\\ & \text{= }{\left(1.96\right)}^{\text{2}}\times 0.355\times (1-0.355)/0.05^{\text{2}}\\ & \text{= }351.85\\ & =352 \end{array}$$

where,

n = calculated sample size
Z = 1.96 at 95% Confidence Interval
p = prevalence of Excessive Daytime Sleepiness taken from previous study (35.5%)^6^
q = 1-p
e = Margin of error (5%)

In NAIHS, students currently studying from 1st to 4th year (N) = 423.

Adjusted sample size (n') = n/ [1+ {(n-1)/N}]

= 352/ [1+ {(352-1)/423}]

= 192.37

= 193

Taking 10% as a non-response rate, the required sample size becomes 213.

Hence, we sent the questionnaire to around 232 students (greater than our required sample size i.e. 213) concerning the fact that some of the students might not give the consent to participate in the study.

Participants were selected using stratified random sampling techniques so that every student from the first to fourth year currently enrolled in NAIHS-COM has an equal probability of being selected in the study. First of all, the list of students from the first to the fourth year was obtained from the administrative section of the institute, and each student was assigned a particular random number. An equal proportion of the students from each year was taken for the study. Similarly, the proportion of males and females from each year was also equal. Then the study participants were selected randomly using the computer method keeping in mind the proportion of students from each year and the proportion of males and females in each year.

We used the standard and validated questionnaire based on the Epworth Sleepiness Scale (ESS), which was developed by Dr. Murray Johns in 1990 and later modified in 1997. The permission to use the questionnaire has been granted to us by the Mapi Research Trust, in France. ESS contained 8 questions for which the study participants are asked to rate on a 4-point scale (0-3) correlating the individual's usual chance of dozing off while engaged in eight different activities mentioned in the questionnaires. The overall score range from 0-24 and Excessive Daytime Sleepiness (EDS) is characterized by the ESS>10. The questions were re-typed in google form after the permission from the respective authority and the form was distributed to the selected study participants online.

Out of 232 students, one refused to give consent and 15 of the students were inaccessible to obtain the data. Hence, we obtained complete data from 216 students whose responses were entered into the Google spreadsheet and later analyzed.

## RESULTS

**Figure 1:**
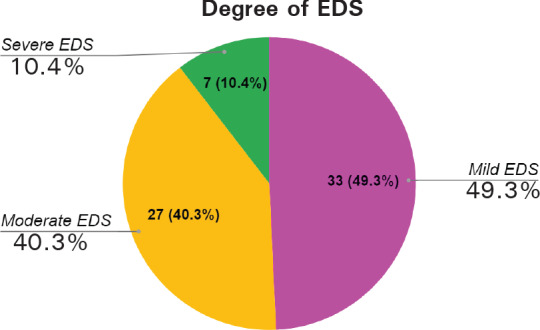
Degree of Excessive Daytime Sleepiness.

The prevalence of Excessive Daytime Sleepiness (EDS) among the first to fourth year undergraduate students of NAIHS-COM is found to be 67 (31.02%) at 95% Confidence Interval (24.85-37.19). ESS characterized mild, moderate, and severe EDS as ESS scores 11-12, 13-15, and 16-24 respectively. In our study, we found mild, moderate, and severe EDS to be 33 (49.25%), 27 (40.30%), and 7 (10.45%) respectively (Figure 1).

EDS was found to be highly prevalent among the fourth year undergraduate medical students and it is least prevalent among the first year students of NAIHS-COM. Numerically, EDS corresponds to 20 (35.09%) in the fourth year followed by 18 (33.33%) in the second year followed by 16 (29.09%) in the third year, and 13 (26.00%) in the first year (Table 1).

**Table 1:** Distribution of Excessive Daytime Sleepiness among first to fourth year.

Year in the medical school	Total number of students selected for the study	Excessive Daytime Sleepiness n (%)
Fourth	57	20 (35.09)
Third	55	16 (29.09)
Second	54	18 (33.33)
First	50	13 (26.00)
Total	216	67 (31.02)

Among the 216 participants, there were 150 males and 66 females. In our study, EDS was found to be slightly higher among females than males which correspond to 23 (34.85%) among females and 44 (29.33%) among males (Table 2).

**Table 2:** Gender wise distribution of Excessive Daytime Sleepiness.

Gender	Total number	Excessive Daytime Sleepiness n (%)
Female	66	23 (34.85)
Male	150	44 (29.33)
Total	216	67 (31.02)

## DISCUSSION

Excessive Daytime Sleepiness refers to the uncontrollable sleepiness during the daytime that leads to the inability of an individual to remain awake in the situations in which the person is reasonably expected to be alert.^2–3^ It is a significant public health problem that impairs daily activities, affects academic and cognitive performances, and can lead to potentially life-threatening road accidents and occupational injuries.^2,7^ Various objective methods such as polysomnography, Multiple Sleep Latency Test (MSLT), Maintenance of Wakefulness Test (MWT), etc. can be used to assess daytime sleepiness. On the other hand, Epworth Sleepiness Scale (ESS) is a widely used subjective method for assessing EDS which is simpler, cheaper, and less time-consuming.^8–9^ ESS was developed by Dr. Murray Johns in 1990 which he named after Epworth Hospital in Melbourne, where he established the Epworth Sleep Centre in 1988. According to ESS, EDS denotes ESS score >10 which is further categorized as mild, moderate, and severe EDS for ESS scores 11-12, 13-15, and 16-24 respectively.^10^ Our study used ESS as a method to determine the prevalence of EDS among the first to fourth year medical students of the Nepalese Army Institute of Health Sciences in Nepal.

There are various studies on EDS conducted among the general population and medical students worldwide. Studies conducted by Boyes J, et al., Wu S, et al., and Drakatos P et al. have found the prevalence of EDS among the general population to be 51.5%,^8^ 22.16%,^9^ and 44.3%^11^ respectively which suggested EDS to be highly prevalent among the general population. On the other hand, a review article by Slater G, et al. has shown that the estimated prevalence of EDS in a “Sleep in America” poll conducted by the American Sleep Foundation is 18% and in a Norwegian study is 17.7%.^12^ These studies showed variable, however, increasing prevalence of EDS among the general population depicting EDS as the widespread health problem in the community. According to these studies, risk factors associated with EDS are poor sleep hygiene, use of modern media during bedtime, elderly population, female gender, obese individual, various sleep disorders, psychiatric disorders such as depression etc.^8–9,11–12^ Similarly, a study by Wu S, et al. have also associated EDS with education emphasizing on the low and high level of education to be a risk factor for EDS. This study has hypothesized that individuals at a high level of education are mostly brain workers, hence more vulnerable to suffer from EDS as a result of insomnia.^9^

According to our study, the prevalence of EDS among medical students of NAIHS-COM is found to be 31.02%. This finding shows that EDS is a common problem among medical students as well and needs to be addressed on time. We propose that the higher prevalence is due to the heavy academic loads among the medical students as the majority of the medical students in Nepal start their academic classes early in the morning till evening and they spend the remaining hours in the libraries or their hostel room studying till late hours. At the same time, other contributing factors could be the use of social media during night, caffeine intake at bedtime, excessive stress, insomnia, etc. These factors eventually compromise their sleep hours and lead to sleep deprivation. Further studies in the same setting are required to determine the actual causes behind EDS. Findings similar to our study can be observed in other studies done among medical students such as Moroccan, Malaysian, Indian, and Brazilian studies which showed the prevalence of EDS to be 36.6%,^4^ 35.5%,^6^ 30.6%, and 39.55%^13^ respectively.

The studies conducted among Sudanese and Pakistani medical students have demonstrated a higher prevalence of EDS than that of our study which corresponds to 68.5%^7^ and 43.5%^14^ respectively. Such high prevalence can be explained in part by the cut off value of ESS score because in these studies EDS is defined as ESS score>8 and ESS score>9 respectively whereas in our study we have used ESS score >10 to define EDS. In the same way, a higher prevalence of 49.49%^1^ is seen in the study by Kaur G, et al. because the researchers have calculated the overall prevalence of EDS among undergraduate dental and medical students while our study has calculated the prevalence among medical students only. On the other hand, studies by Shen Y, et al., Mume CO, et al., and Avanaki SN, et al. reported a lower prevalence of EDS than that of our study i.e. 24.6%,^2^ 11.2%^3^ and 17.9%^5^ respectively. These variations might be due to differences in medical education, lifestyles among medical students, cultural and geographical backgrounds, and methodologies.

Our study further categorized EDS as mild, moderate, and severe as per the criteria given in ESS. According to our findings, the prevalence of mild EDS is 49.25% and that of moderate and severe EDS combined represents 50.75%. Hence, there are slightly higher cases of moderate and severe EDS in our study. These findings from our study are consistent with the findings of studies by Zailinawati et al. and Bokhari NM et al. which also demonstrated a higher prevalence of moderate and severe EDS equivalent to 66.67%^6^ and 80%^14^ respectively. Concluding all these findings, it can be derived that the burden of moderate and severe EDS among medical students is higher. Hence, it is essential to conduct further studies to determine the risk factors associated with such EDS which will ultimately help to manage these conditions properly.

Zailinawati AH, et al.^6^ and Bokhari NM, et al.^14^ in their studies have shown that the prevalence of EDS increases with an increase in the year of study among medical students. Our study also revealed a similar trend except for the prevalence of EDS in the third year which is comparatively lower than that of the second year. The probable explanation behind the finding in these studies is that the burden of study increases from first to final year along with the addition of clinical hours which ultimately compromises the sleep hours at night and leads to EDS. In the case of our study, the reason for the lesser prevalence of EDS in the third year than the second year could be less burden of study as the curriculum in the third year is flexible whereas students in the second year are under high pressure due to increased study loads.

Our study incorporated an equal proportion of males and females through stratified random sampling. There is a slightly higher prevalence of EDS among females than males in our study which is also supported by the results of other studies by Kaur G, et al.,^1^ Shen Y, et al,^2^ Mume CO, et al^3^ and Avanaki SN, et al.^5^ Some studies have associated such higher risk of EDS to psychopathology such as anxiety and depression among female.^2–3^ Similarly, there were studies which showed a much higher prevalence of EDS among females i.e. 63.3%^4^ and 45.3%^14^ which can be attributed to the greater proportion of female included in these studies i. e. 70.7%and 68% of the total sample respectively.

There are certain limitations in our study. As the study is being conducted among the medical students of a single medical college the results can't be generalized to students of other medical colleges of Nepal. Hence, multicenter studies on the EDS can be conducted to improve the generalizability of the findings. Being a descriptive cross-sectional study, our study doesn't determine the causality and risk factors associated with EDS. Further studies are necessary to explore the risk factors associated with EDS among medical students in our setting. There might be certain biases associated with our study such as recall bias as the participants may not be involved in all the activities mentioned in the questionnaire in recent times and subjective bias as the questionnaire is based on the subjective feeling of sleepiness on doing different activities.

## CONCLUSIONS

Excessive Daytime Sleepiness is found to be highly prevalent among medical students of a medical college in Nepal. Further studies are needed in this aspect to determine the risk factors associated with EDS among medical students. It is essential to create awareness among the students to manage EDS properly because various studies have suggested the detrimental effects of EDS in daily activities, cognitive and academic performances.

## Conflict of Interest

**None.**

## References

[1] Kaur G, Singh A (2017). Excessive daytime sleepiness and its pattern among Indian college students. Sleep Med.

[2] Shen Y, Meng F, Tan SN, Zhang Y, Anderiescu EC, Abeysekera RE (2019). Excessive daytime sleepiness in medical students of Hunan province: Prevalence, correlates, and its relationship with suicidal behaviors. J Affect Disord.

[3] Mume CO, Olawale KO, Osundina AF (2011). Excessive daytime sleepiness, nocturnal sleep duration and psychopathology among Nigerian university students.

[4] El Hangouche AJ, Jniene A, Aboudrara S, Errguig L, Rkain H, Cherti M (2018). Relationship between poor quality sleep, excessive daytime sleepiness and low academic performance in medical students. Adv Med Edu Pract.

[5] Avanaki SN, Avanaki NN, Soleimani P, Rafiei H (2018). Prevalence of daytime sleepiness among medical university students. J Prev Epidemiol.

[6] Zailinawati AH, Teng CL, Chung YC, Teow TL, Lee PN, Jagmohni KS (2009). Daytime sleepiness and sleep quality among Malaysian medical students. Med J Malaysia.

[7] Mirghani HO, Ahmed MA, Elbadawi AS (2015). Daytime sleepiness and chronic sleep deprivation effects on academic performance among the Sudanese medical students. J Taibah Univ Med Sci.

[8] Boyes J, Drakatos P, Jarrold I, Smith J, Steier J (2017). The use of an online Epworth Sleepiness Scale to assess excessive daytime sleepiness. Sleep Breath.

[9] Wu S, Wang R, Ma X, Zhao Y, Yan X, He J (2012). Excessive daytime sleepiness assessed by the Epworth Sleepiness Scale and its association with health related quality of life: a population-based study in China. BMC Public Health.

[10] Johns MW (1990). The Epworth Sleepiness Scale [Internet].

[11] Drakatos P, Ghiassi R, Jarrold I, Harris J, Abidi A, Douiri A (2015). The use of an online pictorial Epworth Sleepiness Scale in the assessment of age and gender specific differences in excessive daytime sleepiness. J Thorac Dis.

[12] Slater G, Steier J (2012). Excessive daytime sleepiness in sleep disorders. J Thorac Dis.

[13] Azad MC, Fraser K, Rumana N, Abdullah AF, Shahana N, Hanly PJ (2015). Sleep disturbances among medical students: a global perspective. J Clin Sleep Med.

[14] Bokhari NM, Zafar M (2020). Daytime sleepiness and sleep quality among undergraduate medical students in Sialkot, Pakistan. Dr. Sulaiman Al Habib Medical Journal.

